# Abstracts and Gleanings

**Published:** 1883-12-20

**Authors:** 


### ABSTRACTS AND GLEANINGS.

**Tartar-Emetic in Skin Affections.**—A Surgeon of St. Mary's Hospital (British Med. Jour.) writes:

Tartar emetic, or tartarated antimony, is the preparation I have used in these investigations the largest dose being 1-32 of a grain, or seven and one-half minims of the vinum, only half of the minimum dose of the British Pharmacopœia. I must mention that in all cases in which the effect of the drug has been watched little or no local treatment has been used.

I will state now in as concise a manner as possible, some of the more important diseases in which I have used the drug, leaving a more complete and detailed account for another opportunity.

*Eczema.*—It is now seveıal years since my colleague, Dr. Cheadle, pointed out to me the value of antimony in the treatment of the acute form of this disease. In the majority of the cases which have come under my care, its beneficial effect has been both marked and rapid. In the acute general eczema of adults, which usually commences somewhat suddenly by heat and burning on tha flexor surfaces, and on the other characteristic positions, and is soon followed by abundant exudation of clear fluid, and in the form known as eczema rubrum, I generally begin with four or five minims of the vinum antimoniale three times a day, increasing the dose gradually up to seven minims. After a few doses the exudation ceases, and the local irritation is much relieved; but, in order to prevent a relapse, it is necessary to continue the treatment until all traces of the eruption have disappeared. In acute eczema of children, the dose should be in proportion to the age of the child —half a minim or less up to six months, and one minim or less up to a year. As a rule, I have found both children and adults bear these quantities well, neither sickness or diarrhœa being produced. In the case of aged persons, however, the dose should not exceed three or four minims to begin with, as diarrhœa may result from the administration of a greater amount.

In the subacute forms, both of children and adults, similar doses, but continued for a longer period, are necessary. In chronic eczema, especially when localized, the use of antimony is less often successful; but even in this troublesome form, it relieves the acute exacerbations, and is occasionally followed by cure when other methods of treatment have failed.

In eczema impetiginodes of children, I have noticed little benefit from the drug till the scabs have been removed, and formation of pus checked by local treatment. Simple impetigo contagiosa from a local cause is not included in this category.

In various forms of so-ealled lichen that occur in children, I have found antimony in the previously mentioned doses of the greatest value in relieving the irritation—a feature in which it resembles arsenic.

*Prurigo.*—In this troublesome affection, frequently met with in our out-patient rooms—the relation of which to the severe form known on the Continent as Hebra's prurigo, Mr. Morrant Baker pointed out at the International Congress of 1881—antimony is of great use. Three or four minims of the vinum, continued over a long period, allays the itching to a large extent, and often prevents the relapse of eczema. In several cases, after arsenic, iron, iodide of iron, cod-liver oil and numberless other tonics had been tried, antimony was the only drug that produced any benefit whatever. When given in the before-mentioned doses continuously for more than a year, I have never seen sickness, diarrhœs, sweating, or debility; but, on the contrary, the appetite improves and the weight increases. I have not had the opportunity of trying the remedy in a patient older than 18½ years, suffering from this disease; but in one particular case of that age the benefit was most marked while the drug was being taken.

*Sycosis.*—I have given antimony in five well-marked cases of this disease; in four, it did not seem to produce any effect, either beneficial or otherwise; in the fifth, there was considerable improvement after the vinum had been taken a fortnight in seven-minim doses. It seemed to relieve the pain and burning; but, although the remedy was persevered with for over three months, the improvement was only temporary. The local treatment while the drug was being administered was olive-oil or vaseline. In none of these cases was there any bad effect; no depression, diarrhœa, sickness or sweating.

*Urticaria.*—In a few cases of chronic urticaria, I have found antimony, like arsenic, of service in checking attacks, so long as the remedy was continued.

*Psoriasis.*—Though, in the majority of cases of psoriasis, arsenic is to be preferred to antimony, I have elsewhere called attention to the fact that, in certain persons, arsenic not only fails to relieve, but even aggravates the disease. I have, in some of these cases, tried antimony, and have noticed that in a few instances improvement took place, while in others it seemed to have no effect.

I have been obliged to condense the facts in this paper iuto very brief space, but two points I wish especially to lay stress on: first, that tartar emetic—in doses of 1-240 to 1-32 of a grain, according to age—can not only be tolerated, hut seems to have a decided tonic action; secondly, that it proves useful in those acute forms of skin disease that are usually aggravated by arsenic.

**New Operation for Prolapsus Recti.**—Dr. D'Antona has performed, with success, the following operation on a woman: Seizing the prolapse with four Billroth's pincette's, and forming thus two cylinders of the rectal canal, he introduced one catgut suture into both cylinders and then into the margin of the anus. Another suture is passed through the middle part of one cylinder, carried through the Douglas sac, and the perirectal tissue, returning to the other cylinder. The patient is discharged, cured in fifteen days.—*Med. aud Surg. Rep.*

**The Physician.**—A poem by Hugo Erichsen, read before the Delta-mu Society of the University of Vermont:

Hail to the doctor, on he toils
  In happy and in weary days;
His enemy, grim death, he foils,
  And full of hardships are his ways:

They lead from scenes of agony,
  To scenes of joy and happiness;
From deathbed, which is irony
  Upon the doctor's helplessness,

To cradles filled with human buds,
  Which will become the flowers fair
That fill the world with happiness
  Or scatter poison everywhere.

Within his bosom, hidden, lie
  The secrets by his patient told,
And vainly would a person try
  To make him tell, by force or gold.

He soothes the pain and heals the wounds
  Made by disease or furious foe,
Or by Time's swift pursuing hounds,
  Who bite the men unless they go

Their way is toward the distant end,
  O'er stony path with restless zeal;
Nor can a friend assistance lend
  To keep the hounds from off their heel.

You smile, and don't believe the tale
  Of that wild everlasting race:
Look at the old, for every bite
  There is a furrow in their face.

The book of history is o'er filled
  With names of horoes, crowned with fame,
Because in battle they have killed
  Their fellow-beings: such makes fame.

Not so, the doctor; he saves life,
  And in the fever-stricken land
He shows what heroism is;
  And through disease, with steady hand,

He leads his patient. "All is safe,"
  He finally whispers, glad the more
That he has saved another life.
  "God bless the doctor o'er and o'er."

—*Fort Wayne Med. Jour.*

**Specific Treatment of Typhoid Fever.**—A writer in the Medical Investigator gives the following as the so-called specific treatment of this disease:

In typhoid fever, when the pulse is 90 to 100 soft and small, give aconite, gtts. v to viij, water oz. iv; dose, a teaspoonful every one or two hours. If the pulse is full and hard, use veratrum in place of aconite; if the pulse is medium, full and hard, use aconite and veratrum combined. This will control the circulation, and we can readily bring and keep the pulse down to 75 and 80. This will also keep down the temperature. If there is nervous irritation with pain in the head, papillæ on tongue elevated, add rhus, gtts. x, to the sedative. If the patient is stupid, dull eyes, pupils dilated, add belladonna, gtts. v to x, in the sedative in place of the rhus. If the face is flushed, with determination of blood to the brain, add gtts. xx of gelsemium to the sedative. But remember, never give gelsemium and belladonna combined, as they have a specific distinct action and effect. These remedies, when well selected and properly administered, will have a nice effect in controling febrile excitement.

But perhaps the most important part of treatment in this fever is to neutralize and eliminate the blood poison, and just what that blood poison is, the profession has not clearly determined, but is a blood poison whatever it may be, and the lesion is in the Peyer's glands in the small intestines.

The appearance of the tongue will readily point out the remedies. If the tongue is broad, pallid and white-coated, which is observed in some cases, give five grains of sulphite of soda every three hours, alternating with the sedative. If the tongue appears pale-red, give the nitro-muriatic acid diluted, gtts. v in water every three hours. If the tongue has a purplish-brown color, give baptisia oz. ss. to water oz. iv, a teaspoonful every four hours.

One of these last remedies is specifically indicated in every case and will neutralize the blood poison.

The above is the general outline of specific medication. Of course I would keep the bowels controlled, and kidneys, liver and skin, in good, active condition, and perhaps give some stimulants, with quinine inunction, if indicated.

I could readily explain why these remedies help in curing the disease, but it would take up too much space.

The last five years I have treated a number of typhoid fever patients on this line of treatment, and the result is very satisfactory, and can cheerfully recommend it.

**Glycerine** internally administered (Dr. Tisne, Gaz. des Hop.), exerts a beneficial effect upon nutrition, increasing the weight and palliating many of the distressing symptoms in phthisis, such as loss of appetite, diarrhœa, night-sweats and insomnia. Its action upon the liver is manifested by an increase in the size of the organ and a more abundant flow of bile. It has a diuretic effect, and increases the excretion of urea, the chlorides and the phosphates. The alkalinity of the urine is diminished, and if any pus be present in this it is greatly lessened in amount.—*N. Y. Med. Jour.*

**The Antagonism between Paraldehyde and Styychnine.**—Professor V. Cervello has examined paraldehyde, which he recommended in an earlier contribution as a substitute for chloral hydrate, (and which has since been more thoroughly investigated by Morselli and Albertoni,) in reference to its efficacy in strychnine poisoning. From experiments on frogs and dogs, he comes to the following conclusions:

Animals to which fatal doses of strychnine have been administered may be rescued by non-fatal doses of paraldehyde, which not only prevents death, but also the appearance of the symptoms of strychnine poisoning.

Narcosis by paraldehyde is the same in animals poisoned by strychnine as in those in a normal condition. The temperature and frequency of breathing always become less, and the reflex action weaker.

Paraldehyde overcomes strychnine poisoning in doses not nearly sufficient to produce narcosis. Previous administration of strychnine delays the narcosis of paraldehyde, but its course undergoes no modification.

Paraldehyde hinders the increase of the blood-pressure caused by strychnine.

With frogs, the effects of paraldehyde are but transient, since it quickly passes out of the system; but strychnine remains a longer time, and its effects are more lasting.

Poisoning by paraldehyde is not antagonized, and in general is not modified, by strychnine, either in large or small doses. No mutual antagonism exists, therefore, between the two poisons. Both act centrally—one augmenting, the other diminishing, the reflex action of the gray substance of the medulla.—*Translation from the Deutsche Medizinal-Zeitung, October 4, 1883, in The Polyclinic.*

**Croup and Diphtheria.**—G. W. Church, M. D., Shaftsburg, Mich., writes: In the July 10th (1883) number of the Age, in reporting the transactions of the American Medical Association, I notice remarks on "Unity of Diphtheria and Membranous Croup" —a paper read at the Association by Dr. A. Harris, of Virginia.

In reference to this subject, it probably is generally known that very many physicians throughout the country hold the views of Dr. Harris, viz: that these two diseases are not two, but one and the same—identical. If so, how shall we account for the difference in morbid anatomy and symptomatology?

1. Croup is sporadic; diphtheria is contagious.

2. Croup is non-contagious; diphtheria is contagious.

3. The pseudo-membrane in croup is strictly *upon* the mucous membrane; in diphtheria it is not only upon, but infiltrated and sub-mucous.

4. In croup we have to deal with a local disease, in diphtheria with a constitutional.

5. In croup we have causation: (*a*) constitutional tendency, (*b*) vicissitudes of temperature, (*c*) the inhalation or swallowing of irritants; in diphtheria (these do not act as causes), exposure to the *materies morbi* only.

6. The pseudo-membrane in croup has, I believe, never been known to invade other parts; in diphtheria it may be found on almost any delicate surface, as the lining of the external ear, the vagina, under the prepuce, conjunctiva, stomach, and on the cutaneous surface, if denuded or cut.

7. The most important difference is found in the state of the blood, after death.

It would seem that the idea of their unity was drawn from coincidences—the pseudo-membrane in both, and the cynanche (not always present in diphtheria, but always in croup, and the principal feature in the case).

As to the nuropathic elements of the two diseases, the profession are as little agreed as to their unity.

Paralysis of the laryngeal apparatus does not account for the symptoms, and the idea seems borrowed from diphtheria. Spasm seems to fully account for these symptoms. Do not our conclusions get the start, sometimes, of our reason ?—*Med. Age, July 25.*—*Fort Wayne Med. Jour.*

**Treatment of Typhoid Fever.**—Dr. Delafield (in New York Medical Record) says:

"A fair idea of the manner in which typhoid fever is treated in New York may be gathered from the routine of the different hospitals.

In the New York Hospital many patients are simply put on a milk diet, with the addition of a moderate amount of whiskey, and no other treatment is used. Peptonized milk instead of ordinary milk is thought to be of service. For high temperatures the body is sponged with equal parts of alcohol and water, and sometimes the fluid extract of eucalyptus is given in fifteen-minim doses. Quinine is not much used. Tympanites is treated with turpentine internally, and in stupes over the abdomen. Opium is given when there is hemorrhage from the bowels or excessive diarrhœa.

At St. Luke's Hospital the treatment is the same, except that quinine is sometimes employed to reduce the temperature, and ergotine hypodermically for intestinal hemorrhage. Either opium or chloral are used to control restlessness and sleeplessness.

At St. Francis' Hospital, if the cases are seen early in the disease, large doses of calomel are given, with the idea of aborting the disease. Quinine in large doses is given to most of the patients. The salycilate of soda or the benzoate of soda are given by some of the physicians throughout the disease. Cold water in any form, to reduce the temperature, is but very little used. A solution of the acetate of alumina is given to nearly all the patients to prevent or control the diarrhœa.

At St. Vincent's Hospital quinine in doses of two grains every two hours is given to control the temperature. Cold water is not employed. Opium is used with diarrhœa and intestinal hemorrhage.

At the Mount Sinai Hospital quinine in large doses is given to nearly all the patients. Cold water is not much used, but sometimes the patients are sp·nged off.

At Bellevue Hospital the treatment varies in the different divisions.

In one division, the peptonized milk is much used. Quinine, in large doses, is given when the temperature reaches 103°, and sponging is also sometimes used. Opium, the bromides, and cold to the head are used for the restlessness.

In another division quinine in moderate doses is given to most of the patients. For temperatures over 103° sponging with cold water or the Kibbee cot and sprinkling with cold water are used. Opium is given when needed.

In another division carbolic acid, gtt. j, and tincture iodine, gtt. ij, every two hours, are given early in the disease. Quinine in ten-grain doses every half hour is given to reduce the temperature. Sponging with cold water is sometimes used. Opium is employed for severe diarrhœa.

In another division occasional sponging, and whiskey and opium when required, are the only treatment.

At the Roosevelt Hospital full bathing has been tried in many cases, but now cold sponging is more used. Bismuth and pepsin are given to many of the patients.

In all the hospitals milk, either simple or peptonized, is the regular diet of the patients.

**Benzoate of Iodine** is recommended by Dr. W. P. Watson, in the Medical Record, in the treatment of acute gastro-intestinal diseases—dysentery, cholera morbus, colic and the like. The philosophy of its action he does not undertake to formulate but has found it to give quick and permanent relief. In a casc of dysentery reported, where the symptoms were quite urgentt and had failed of relief by various diarrhœa mixtures domestically given, relief was marked at the first dose, vomiting and tenesmus being soon entirely restrained and the discharges becoming feculent. It was given in doses of seven and a half grains, every half hour, in simple elixir, until four doses were taken. In three similar cases it proved satisfactory.

In a case of cholera morbus fifteen grains were given at once and half that quantity repeated at half-hour intervals, with complete relief in a few hours. The same treatment was used in a case of severe intestinal colic, where morphia was known to have had bad after effect, was attended with similar results, relief coming within a few minutes after the first exhibition of the remedy. It has also been found useful in the bowel complaints of children when the passages are greenish, lumpy, mucoid and frequently streaked with blood, giving it in hourly doses of one grain for each year of age of the child.—*Med. Annals.*

**Auto-Transfusion in Hemorrhage.**—Attention has been quite frequently called to the value of auto-transfusion in cases of severe hemorrhage. In the *Wiener Med. Blatter*, of February 23d, Professor Braun relates a case of inversion of the uterus in parturition with severe and almost fatal hemorrhage, in which this method was resorted to. Elastic bandages were wrapped around the legs, with an immediate dissipation of the alarming symptoms. The hips were raised, ether injected, and stimulants administered. The bandages were kept on for nineteen hours. This method is especially applicable to post-partum hemorrhage from any cause, and is easily and quickly applied, much more so than transfusion. Doubtless, so prolonged an application might have its dangers in a few instances, but the advantages would far more than counterbalance the dangers. It may be remarked that the inversion was caused by pulling on the cord of an adherent placenta, a far too frequent error, we fear, in view of the dangerous results which have, in many instances, followed it.—*N. Y. Med. Rec.*

**Syphilitic Inuoculatiou of Monkeys.**—At a recent meeting of the Societe Medicale des Hopitaux, Dr. Martineau announced that the monkey he inoculated on November 16, 1882, with syphilitic matter from a patient of his, after having presented the characteristic lesions—hard chancre, followed by the various syphilides (papulo-erosive)—became affected ten months after inoculation with ulcerous syphilide of the mucous membrane of the palate, which has healed. This case is interesting in so far as it proves that the evolution of syphilis in this monkey is following the usual course observed in man, and will tend to affect the theories in vogue respecting the natural history and origin of syphilis. —*N. Y. Med. Rec.*

**Cascara Amarga.**—Dr Tangeman reports, in Therapeutic Gazette, the following case treated with Cascara Amarga: Henry K., aged 28, German, carpenter by occupation, made application for the relief of a chancre, which was about the size of a large pea, situated on the prepuce. Patient was very anæmic (though formerly quite healthy), weak, and covered with a pustular eruption. The lymphatic system was involved; some of the glands were enlarged, especially those in the groin, amounting to a bubo on one side. Patient was put on cascara amarga at once, to the exclusion of all other remedies, one teaspoonful three times a day. Improvement was marked; the eruption now disappeared, the bubo did not go to suppuration, and the initial lesion healed rapidly.

But here comes the most interesting portion of the case: The appetite that was wholly lost returned, bowels that were constipated acted very nicely under the influence of this drug, and the patient, in the course of a week, felt buoyant. Altogether, that one case consumed about one pound of cascara amarga. In three weeks he was discharged as cured. During the whole course of treatment there was not a single complaint that the medicine was affecting him badly, which is frequently the case where mercury is given, and often long before your case is ready to be discharged. It is a very mild remedy, and in reality should be prescribed prior to the employment of any other specific remedies, instead of being resorted to only when our old stand-bys fail to relieve.

Case 2 was one of gonorrhœal rheumatism, where the patient had suffered all the pain peculiar to this disease for months. He was a sporting man and had led a sort of a dissipated life, though he was not broken down in strength. He had tried all the remedies, both patent and regular, he claimed, without a particle of relief, not even as much as to relieve the pain sufficiently to obtain one single night's sleep; the seat of the trouble was mainly in the knees and ankles; they were sensitive to the touch. Such was, in short, the patient's condition when he made application for relief. The firm of Parke, Davis & Co. had supplied me a sample of the above drug, and although it was a little out of my domain, I concluded to embrace the opportunity and determine the effects of amarga in this most tedious trouble, as those will know who have ever attempted to treat gonorrhœal rheumatism.

℞ Cascara amarga (P., D. & Co.), } aa..............℥ ij.

Jamaica dogwood, }

M. Sig.

One teaspoonful every three hours in a little water was prescribed and the patient discharged with the request to return and repoit the results. Five days later he returned, and from the smile on his face I could readily read his condition; he could walk very much better, had slept soundly every night since he commenced taking the medicine, and the fact that is to be emphasized is that his stomach was in good condition, which is more than ever can be said for mercury and iodide of potassium. The same prescription was given as before, and patient took no more medicine; he, himself, was surprised at the result, since he had swallowed all the nostrums that had ever been advertised to have any specific effects.

**Petroleum in Phthisis.—Consumption.**—The almost hopelessness of phthisis pulmonalis in the most ordinary cases behooves us to be on the lookout for even a palliative. One theory after another is swept away. Pathological investigation of the most minute microscopical kind fails to detect the cause of this dread disease. The discovery of Koch led us to hope that the cure of it was in our reach, but his supposed discovery of the bacillus has been proved fallacious by other investigators. We are as much in the dark in regard to the ultimate cause of tubercle as ever.

Laying all theory and scientific investigation aside, what can be done in the way of treatment?

Reading an article several years ago by Dr. M. M. Griffith, of Bradford. Pa. (oil country), in regard to the use of petroleum mass in phthisis and chronic bronchial diseases, I have ever since followed his plan and with the most gratifying results:

CASE I. A wood-chopper; complete dullness on left upper lobe of lung; no expectoration; hacking, irritative cough; good appetite; night sweats. He did not think there was anything wrong with his lungs; he thought something was wrong with him, didn't know what; had no fever; pulse and temperature somewhat above normal.

I gave him a box of petroleum pills, four or five pills daily, and to paint the left lung night and morning with tincture of iodine, to remain in the pine woods and report every two weeks.

He followed my directions promptly, and after three months treatment I fail to find any remaining dullness, or cavity, respiratory murmur perfectly clear, night sweats entirely gone. To all appearance he is cured. He states that "he can peel as much bark and chop as many logs as ever." The deposit seemed to be absorbed without cough or expectoration. There was no hereditary taint in his family.

CASE 2. Mr. J. B., aged thirty-nine, fair skin, sandy hair, blonde, occupation miner, family history good, called to see me December 1st, 1881. For a year he had pain in his chest, cough, expectoration and chills. Emaciation of body. Pulse 100, standing; respiratory murmur increased; slight dullness on percnssion of the left upper part of lung; pain sharp, with tenderness on pressure and percussion; cough irritated the larynx; expectoration tough, difficult, brownish, about two ounces per day. Had chills, with hectic fever following, dyspnœa and palpitation, skin dry and harsh. This was plainly a case of phthisis pulmonalis.

He was given petroleum pills, one five times per day, counter irritation over diseased lung. R. Tincture calisaya, iron and strychnia. Teaspoonful tet die.

The first time I saw him after prescribing was one month. The improvement was considerable, cough and expectoration diminished, appetite fair, pain in lung did not trouble him much; stopped the tonic, continue the pills and use no other medicine.

In six months from December 1st he writes me that his health is beter than for years. He has resumed his work in the mines and feels strong and well.

At this date he writes me that his old trouble has not returned. I think this was a genuine case of consumption cured. I have a list of fifteen cases of what I consider true phthisis—ten of which were to all intents and purposes cured, three very much benefitted and two died. It is not only in phthisis that the petroleum acts beneficially, but in all old chronic bronchial diseases. I have nothing new to offer more than has been said by others. I am sure this remedy is not receiving the attention it should by the profession. The dose usually prescribed is too large. The mass is the most eligible form of administration. I have very little experience with the oil, do not use it at all, the pill form being the most convenient and eligible.—*M. Milton, M.D., in ths Cincinnati Lancet and Clinic.*

**Treatment of Puerperal Convulsions.**—Dr. Newcomer, (in Weekly Medical Record) says:

I have met six cases of eclampsia, all of which were bled largely except one. I will not tire you by giving the cases in detail, but simply say that four of them were primipara, and the other two had borne children before. In two the attacks came on during labor; I bled them freely, gave chloroform and hastened the delivery; with the completion of labor the convulsions ceased. In two (primipara) the attacks came on about the eighth month of gestation ; came on without any premonition, or labor pains, or dilatation of the os. I bled them profusely, and kept them under the influence of chloroform four to six hours, and gave them bromide of potass.; did not interfere with pregnancy ; in about six days they were taken with labor pains, and still-born children were expelled, without any return of convulsions ; in the other two the convulsions came on after the completion of labor, one of which had only one spasm and yielded readily to the soothing influence of bromide of potass. and chloral, the other one had twenty-one spasms before I got to see her. I bled her severely, after which I kept her under the influence of chloroform for about six hours, after which she had no more convulsions ; her tongue was so badly bitten and swollen that it protruded from her mouth for several days, which gave her a frighiful appearance. She was discharged well in about ten days.

My experience, as I have said before, has not been large in the treatment of eclampsia, yet I cannot complain of my success in its management. I would not bleed an anæmic patient with eclampsia, but I have never met with such a one. My cases were all quite healthy with a tendency to plethora. Dr. Ohr, in the article formerly referred to, gives an account of over thirty cases of eclampsia treated by himself, with a mortality of two ; one of these was moribund when he first saw her. She was not bled, and the only one of all his cases that was not bled ; this is decidedly the best showing of any of the authors that I have consulted. I feel quite sure that I have averted, on several occasions, attacks of eclampsia by timely bleeding, and yet, after all, we cannot always tell how much or how little good we do. A recent writer observes that "it requires a very accommodating conscience to ascribe every fatal case to circumstances over which we have no control, and to attribute successful cases to our interference." I would not say that my treatment of eclampsia is better than ethers, but as long as it is successful in my hands, I feel justified in continuing it, and recommending it to others.

**Hot Milk as a Restorative.**—Milk that is heated to much above 100° F., loses, for the time, a degree of its sweetness and its density ; but no one fatigued by over-exertion of body and mind, who has ever experienced the reviving influence of a tumbler of this beverage, heated as hot as can be sipped, will willingly forego a resort to it because of its having been rendered somewhat less acceptable to the palate. The promptness with which its cordial influence is felt is indeed surprising. Some portions of it seem to be digested and appropriated almost immediately ; and many who fancy they need alcoholic stimulants when exhausted by labor of brain or body, will find in this simple draught an equivalent that shall be abundantly satisfying, and more enduring in its effects.—*Pop. Sci. News.*

**New Code.**—At the meeting of the New York County Medical Society, on the evening of the 22d ult., occurred the first clean and above-board test of strength in New York City between the

New Code and the Old Code adherents. In previous contests on this question strategy and tricks largely influenced the result, but at the annual election on the 22d the issue was clearly made. The Old Code men had a very considerable advantage as regards their candidate for the Presidency—Dr T. Gaillard Thomas, a gentleman of great popularity and of rare personal magnetism. The vote on officers was, however, clearly understood to be on a question of measnres and not men, and when the ballots were counted it was found by the election of Dr. Van der Poele, that the New Code was sustained by a majority of 155—Dr. Thomas receiving 220 votes and Dr. Van der Poele 375. This very decided vote very effectually settles the question of professional sentiment, outside of the specialists, on the New Code in New York City.

The next and probably final struggle will be at the meeting of the State Medical Society in the coming February. The result of that struggle is no longer very doubtful.—*Med. Age.*

**Hæmoptysis.**—Dr. Brown says: Of drugs, ergot seems to be the most powerful in checking hæmoptysis. The extractum ergotæ fluid may be given in doses of a teaspoonful every fifteen minutes, until the hemorrhage is stopped, and then continued in smaller doses, or it may be given by hypodermic injection, in doses of fifteen drops, or ergotine may be used. If the stomach is irritable, ergotine may be given per rectum. Sometimes ergot will have no appreciative effect. Upon such circumstances I think that gallic acid is the next best remedy. I frequently combine it with aromatic sulphuric acid, which makes a more efficient and pleasant mixture:

℞ Acidi gallici........................................2 ʒ,
Acidi sulphurici aromat...........................1 ℥,
Glycerine...............................................1 ʒ,
Aquæ— q. s. ut. ft...................................4 ℥
M. Sig. A tablespoonful as required.

This is to be given every hour, every half hour, or at shorter intervals, until the hemorrhage is brought under control. This, I think, ranks next to ergot, and where ergot produces no effect I usually resort to this combination.—*Med. Brief.*

**To Remove Fish Bones from the Throat.**—To remove fish bones from the throat, Prof. Voltolina, at Breslau, recommends a gargle composed of muriatic acid, four parts; nitric acid, one part; and water, two hundred and forty parts. The teeth have to be protected by lard or oil. The fish bones become flexible, and they disappear entirely after a short time.—*Med. and Surg. Rep.*

**Small-pox.—Petroleum as an Ectrotic.**—Dr. Kannenski states that he has obtained excellent results, even in the confluent form of small-pox, by painting the skin with a solution of petroleum in olive oil, one to three or four.—*Przeglad Lekarski.—Therap. Gaz., July 16.*

